# Pooled analysis of genome-wide association studies of cervical intraepithelial neoplasia 3 (CIN3) identifies a new susceptibility locus

**DOI:** 10.18632/oncotarget.9916

**Published:** 2016-06-07

**Authors:** Dan Chen, Stefan Enroth, Han Liu, Yang Sun, Huibo Wang, Min Yu, Lian Deng, Shuhua Xu, Ulf Gyllensten

**Affiliations:** ^1^ Ministry of Education and Shanghai Key Laboratory of Children's Environmental Health, Xin Hua Hospital Affiliated to Shanghai Jiao Tong University School of Medicine, Shanghai, China; ^2^ Department of Immunology, Genetics and Pathology, Science for Life Laboratory Uppsala, Uppsala University, Uppsala, Sweden; ^3^ Laboratory of Biochemistry and Molecular Biology, School of Life Science,Yunnan University, Kunming, China; ^4^ Department of Neurosurgery, First Affiliated Hospital of Nanjing Medical University, Nanjing, China; ^5^ Chinese Academy of Sciences (CAS) Key Laboratory of Computational Biology, Max Planck Independent Research Group on Population Genomics, CAS-MPG Partner Institute for Computational Biology (PICB), Shanghai Institutes for Biological Sciences, CAS, Shanghai, China; ^6^ University of Chinese Academy of Sciences, Beijing, China; ^7^ School of Life Science and Technology, Shanghai Tech University, Shanghai, China; ^8^ Collaborative Innovation Center of Genetics and Development, Shanghai, China

**Keywords:** cervical intraepithelial neoplasia 3, genome-wide association study, genetic variants, expression quantitative trait locus, human leukocyte antigen

## Abstract

Recent genome-wide association studies (GWASs) in subjects of European descent have identified associations between cervical cancer risk and three independent loci as well as multiple classical human leukocyte antigen (HLA) alleles at 6p21.3. To search for novel loci associated with development of cervical cancer, we performed a pooled analysis of data from two GWASs by imputing over 10 million genetic variants and 424 classical HLA alleles, for 1,553 intraepithelial neoplasia 3 (CIN3), 81 cervical cancer and 4,442 controls from the Swedish population. Notable findings were validated in an independent study of 961 patients (827 with CIN3 and 123 with cervical cancer) and 1,725 controls. Our data provided increased support for previously identified loci at 6p21.3 (rs9271898, *P* = 1.2 × 10^−24^; rs2516448, 1.1 × 10^−15^; and rs3130196, 2.3 × 10^−9^, respectively) and also confirmed associations with reported classical HLA alleles including HLA-B*07:02, -B*15:01, -DRB1*13:01, -DRB1*15:01, -DQA1*01:03, -DQB1*06:03 and -DQB1*06:02. In addition, we identified and subsequently replicated an independent signal at rs73730372 at 6p21.3 (odds ratio = 0.60, 95% confidence interval = 0.54–0.67, *P* = 3.0 × 10^−19^), which was found to be an expression quantitative trait locus (eQTL) of both *HLA-DQA1* and *HLA-DQB1*. This is one of the strongest common genetic protective variants identified so far for CIN3. We also found HLA-C*07:02 to be associated with risk of CIN3. The present study provides new insights into pathogenesis of CIN3.

## INTRODUCTION

Worldwide, cervical cancer is the fourth most common cancer in women [1]. Persistent infection with carcinogenic human papillomavirus (HPV) strains is the main cause of cervical cancer and its precursor lesions, cervical intraepithelial neoplasia (CIN) [2]. More advanced lesions, designated CIN3, are considered to be the same as carcinoma *in situ* (CIS) or Stage 0 cervical cancer. Although both population screening and a prophylactic vaccine are available, cervical cancer continues to be a major threat to female health globally. Therefore, further understanding of the contribution of genetic susceptibility to persistent HPV infection as well as tumorigenesis is important for preventing the disease.

We recently performed the first genome-wide association study (GWAS) of cervical precancer (CIN3) in a Swedish population and confirmed previously reported associations with classical human leukocyte antigen (HLA) alleles, including HLA-B*07:02, -DRB1*13:01, -DRB1*15:01, -DQA1*01:03, -DQB1*06:03 and -DQB1*06:02. In addition, we identified three novel loci in the major histocompatibility complex (MHC) region at 6p21.3, rs9272143 between *HLA-DRB1* and *HLA-DQA1*, rs2516448 adjacent to the MHC class I polypeptide-related sequence A gene (*MICA*) and rs3117027 at of *HLA-DPB2*, which acted independently of classical HLA alleles [3].

The statistical power of individual GWAS has been limited by the modest effect size of genetic variants and the number of variants that could be studied. To identify additional cervical precancer susceptibility loci, we expanded the present GWAS by pooling its data with the data from another GWAS, together providing data on 1,553 cases with CIN3, 81 cases with cervical cancer and 4,442 controls, all from the Swedish population. To bring genotype data obtained from different single-nucleotide polymorphism (SNP) arrays into a common framework and provide information on ungenotyped genetic variants, we imputed >10 million genetic variants and 424 classical HLA alleles, using the 1000 Genome Project data and the Type 1 Diabetes Genetics Consortium (T1DGC) panel as reference. We then conducted a comprehensive examination of the association between CIN3 risk and common variants, rare variants as well as classical HLA alleles. Notable findings were validated in an independent study of 961 cases (827 with CIN3 and 123 with cervical cancer) and 1,725 controls.

## RESULTS

### Genome-wide association results

After stringent quality control (QC), data from 1,634 cases (1,553 CIN3 and 81 cervical cancer) and 4,442 controls were available for 5,471,179 SNPs with an overall call rate of 99.32%. The pooled analysis showed modest evidence of over-dispersion (λ = 1.08). Genetic variants mapping to the previously identified susceptiblity loci at 6p21.3 provided the best evidence for an association with cervical precancer (Supplementary Figure S1). A total of 1,576 SNPs showed evidence for an association with CIN3 risk at *P* = 5 × 10^−7^, 1,575 of which are located within the MHC region at 6p21.3 and one of which is located at 18q12.3. After excluding the genetic variants in the extended MHC region where extensive LD extends over long distances, λ is only 1.02 (Supplementary Figure S1). The strongest association was attained for rs9271898 (odds ratio [OR] = 0.64, 95% confidence interval [CI] = 0.59 – 0.70, *P* = 1.2 × 10^−24^ for the minor allele A), which is located between *HLA-DRB1* and *HLA-DQA1* and is highly correlated with rs9272143 (*D’* = 0.99, *r*^2^ = 0.98), the top hit previously reported (Table 1). A borderline genome-wide significant association was observed between CIN3 risk and a locus outside MHC region, that is rs73000875 at 18q12.3 (OR = 0.61, 95% CI = 0.50-0.74, *P* = 3.8 × 10^−7^ for the minor allele G).

**Table 1:** Summary of genome-wide association study results of 4 SNPs at 6p21.3 independently associated with CIN3

					MAF		Single SNP^c^		Stepwise conditional analysis	
Loci	SNP	Position^a^	Nearby gene	Alleles^b^	Case	Control	OR (95%CI)	*P*	OR (95%CI)	*P*
1	rs9271898	32595972 (class I)	*HLA-DRB1, HLA-DQA1*	G > A	0.38	0.49	0.64(0.59–0.70)	1.2×10^−24^	–	–
2	rs2516448	31390410 (class I)	*MICA*	C > T	0.58	0.50	1.39(1.28–1.52)	1.1×10^−15^	1.41(1.30–1.54)^d^	5.6 × 10^−16 d^
3	rs3130196	33063219 (class II)	*HLA-DPB1, HLA-DPA1*	T > C	0.17	0.13	1.40(1.26–1.57)	2.3×10^−9^	1.40(1.25–1.57)^e^	9.4 × 10^−9 e^
4	rs115625939	32583584 (class II)	*HLA-DRB1, HLA-DQA1*	A > G	0.09	0.15	0.58(0.51–0.67)	1.4×10^−15^	0.68(0.58–0.79)^f^	3.2 × 10^−7f^

MAF, minor allele frequency; OR, odds ratio; CI, confidence interval; *P*, two-sided *P* value corresponding to the OR.

a GRCh37/hg19 Assembly.

b Major allele > Minor allele

c ORs and 95%CIs for minor allele in log-additive model were derived from logistic regression with adjustment for study and one significant principal component generated by principal components analysis.

d Conditioning on rs9271898.

e Conditioning on rs9271898 and rs2516448

f Conditioning on rs9271898, rs2516448 and rs3130196.

Concordance between the genotyped and imputed data was 0.96 for *HLA-A*, 0.97 for *HLA-B*, 0.92 for *HLA-DRB1*, 0.92 for *HLA-DQB1* and 0.91 for *HLA-DPB1*, at two field level of HLA typing, respectively. As shown in Table 2, three HLA class I alleles showed evidence of association with CIN3 at the 5 × 10^−7^ threshold, namely HLA-B*07:02 (OR = 1.41, 95% CI = 1.27–1.57, *P* = 1.4 × 10^−10^), HLA-B*15:01 (OR = 0.67, 95% CI = 0.58 – 0.77, *P* = 2.6 × 10^−8^) and HLA-C*07:02 (OR = 1.37, 95% CI = 1.24–1.52, *P* = 2.6 × 10^−9^). HLA-B*07:02 and HLA-C*07:02 are in strong LD with each other (*r*^2^ = 0.87, *D’*=0.96 in controls). Five HLA class II alleles were associated with risk of CIN3 namely HLA-DRB1*13:01 (OR = 0.49, 95% CI = 0.40–0.59, *P* = 1.8 × 10^−13^), HLA-DRB1*15:01 (OR = 1.36, 95% CI = 1.22 – 1.51, *P* = 9.7 × 10^−9^), HLA-DQA1*01:03 (OR = 0.49, 95% CI = 0.40–0.59, *P* = 5.6 × 10^−14^), HLA-DQB1*06:03 (OR = 0.54, 95% CI = 0.45-0.64, *P* = 1.5 × 10^−11^), and HLA-DQB1*06:02 (OR = 1.32, 95% CI = 1.19-1.47, *P* = 2.5 × 10^−7^). HLA-DRB1*15:01 and HLA-DQB1*06:02 (*r*^2^ = 0.95,*D’* = 0.99 in controls), as well as HLA-DRB1*13:01, HLA-DQA1*01:03 and HLA-DQB1*06:03 are in strong LD with each other (*r*^2^ = 0.96 and *D’*= 0.99 between DRB1*13:01 and DQA1*01:03; *r*^2^ = 0.93, *D’*= 0.99 between DRB1*13:01 and DQB1*06:03; r^2^ = 0.91, *D’* = 0.96 between DQA1*01:03 and DQB1*06:03 in controls), respectively (Supplementary Table S1).

**Table 2:** Analysis of possible confounding of the classical HLA allele associations by SNPs

	Allele frequency		Original results^a^		Conditioning on 4 SNPs^b^	
	Case	Control	OR (95%)	*P*	OR (95%)	*P*
**HLA-B**						
HLA_B*07:02	0.20	0.15	1.41(1.27–1.57)	1.4 × 10^−10^	1.08(0.96–1.22)	0.20
HLA_B*15:01	0.08	0.12	0.67(0.58–0.77)	2.6 × 10^−8^	0.76(0.65–0.89)	5.1 × 10^−4^
**HLA-C**						
HLA_C*07:02	0.20	0.16	1.37(1.24–1.52)	2.6 × 10^−9^	1.06(0.94–1.19)	0.38
**HLA-DRB1**						
HLA_DRB1*13:01	0.04	0.08	0.49(0.40–0.59)	1.8 × 10^−13^	0.81(0.62–1.04)	0.10
HLA_DRB1*15:01	0.20	0.15	1.36(1.22–1.51)	9.7 × 10^−9^	1.01(0.90–1.14)	0.84
**HLA-DQA1**						
HLA_DQA1*01:03	0.04	0.08	0.49(0.40–0.59)	5.6 × 10^−14^	0.76(0.59–0.98)	0.03
**HLA-DQB1**						
HLA_DQB1*06:03	0.05	0.08	0.54(0.45–0.64)	1.5 × 10^−11^	0.89(0.70–1.13)	0.35
HLA_DQB1*06:02	0.19	0.15	1.32(1.19–1.47)	2.5 × 10^−7^	0.98(0.86–1.10)	0.69

CI, confidence interval; HLA, human leukocyte antigen; SNP, single-nucleotide polymorphism; OR, odds ratio; P, two-sided P value corresponding to the OR.

a Derived from logistic regression in log-additive model with adjustment for study and one informative eigenvector generated by principal components analysis.

b Derived from logistic regression in log-additive model conditioning on rs9271898, rs2516448, rs3130196 and rs73730372 with adjustment for study and one significant principal component generated by principal components analysis.

**Figure 1:**
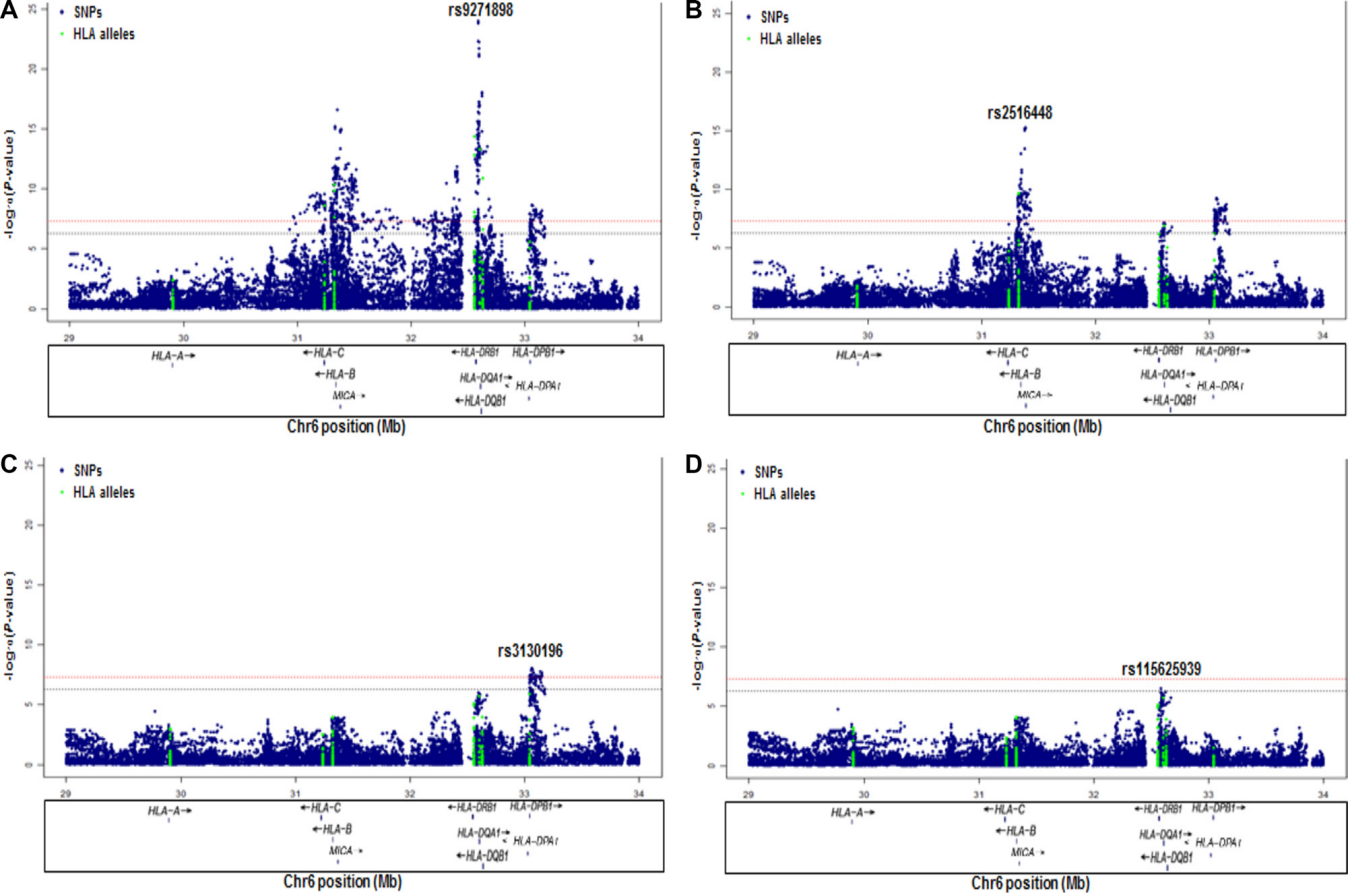
Association results of stepwise conditional logistic regression analysis for CIN3 at 6p21.3 The -log10 (*P* values) for each SNP (y-axis) are plotted against their position (x-axis) on chromosome 6 (hg19). The horizontal red line and blue line represent *P* = 5 × 10^−8^ and *P* = 5 × 10^−7^, respectively. (**A**) Association results in unconditional logistic regression analysis. (**B**) Association results upon conditioning on rs9271898. (**C**) Association results upon conditioning on rs9271898 and rs2516448. (**D**) Association results upon conditioning on rs9271898, rs2516448 and rs3130196.

**Figure 2:**
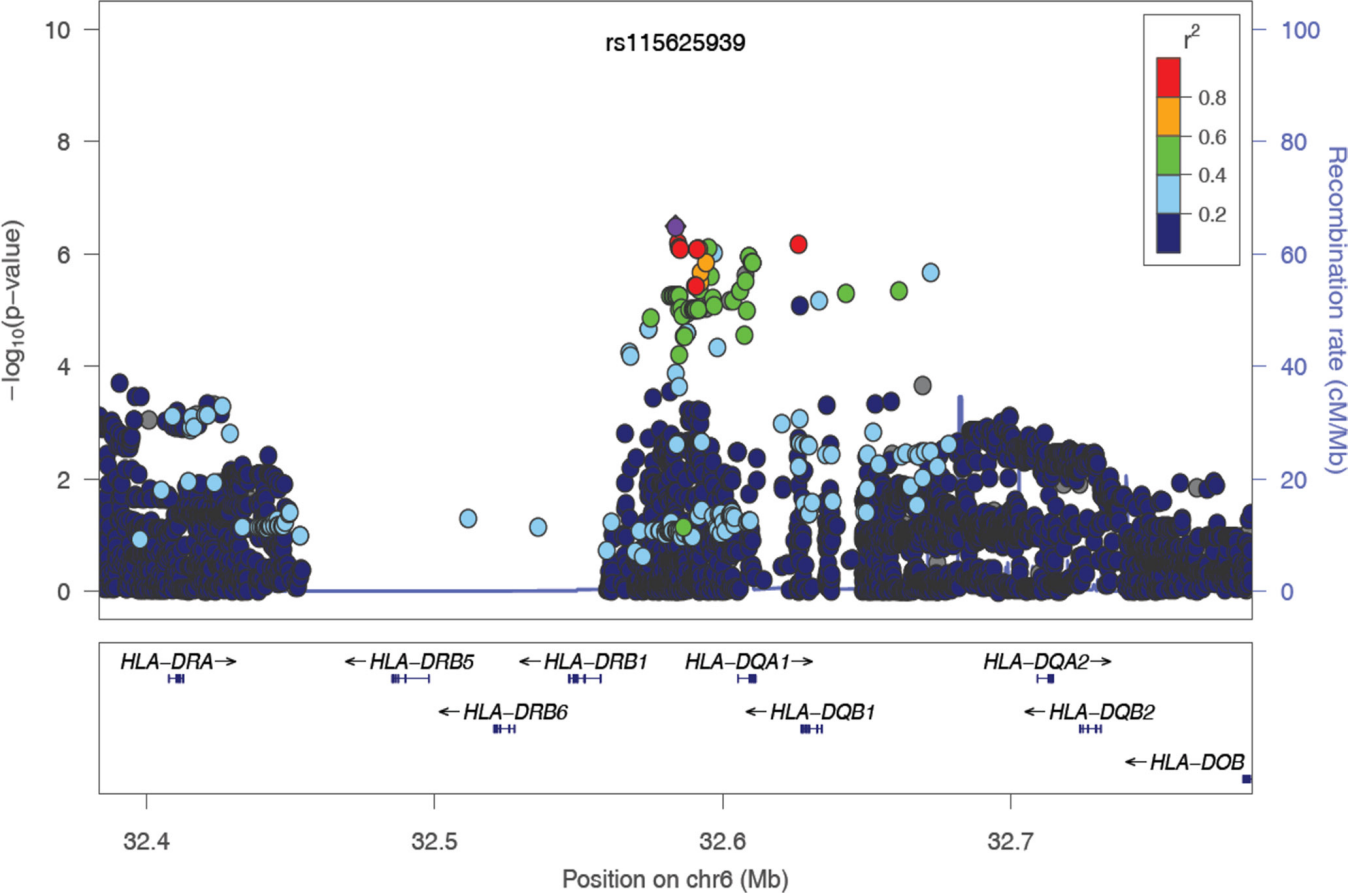
The regional association plot of rs115625939 in the MHC region Results are shown for SNPs in the region flanking 200 kb on either side of rs115625939 upon conditioning on the top 3 hits. The purple circle indicates the top SNP in each locus and the color of the dots represents the degree of linkage disequilibrium (based on *r*^2^) in relation to the top SNP. Recombination rates (cM/Mb) overlay the plots and are based on HapMap data http://hapmap.ncbi.nlm.nih.gov/downloads/recombination/2008-03_rel22_B36/rates/. Genes in the region are represented with arrow heads indicating the direction of transcription.

To evaluate the independence of associations at 6p21.3, we conducted stepwise logistic regression analysis at 6p21.3 (Figure 1, Table 1, Supplementary Figure S2). Consistent with previous findings, after conditioning on the top signal rs9271898, the strongest secondary signal appeared to be for rs2516448 (OR = 1.41, 95% CI = 1.30 – 1.54, *P* = 5.6 × 10^−16^ compared with OR = 1.39, 95% CI = 1.28 – 1.52, *P* = 1.1 × 10^−15^ for the minor allele T for the unconditional analysis), which is located downstream of *MICA*. When further conditioning upon rs2516448, residual association was detected at some SNPs and HLA alleles in this region, with the most significant one occurring at rs3130196 (OR = 1.40, 95% CI = 1.25–1.57, *P* = 9.4 × 10^−9^ compared with OR = 1.40, 95% CI = 1.26–1.57, *P* = 2.3 × 10^−9^ for the minor allele C for the unconditional analysis), which is in LD with rs3117027 (*D’* = 0.96, *r*^2^ = 0.32) and is located downstream of *HLA-DPB1* and upstream of *HLA-DPA1*. Conditioning on all three SNPs jointly still left residual association at rs115625939 (OR = 0.68, 95% CI = 0.58–0.79,*P* = 3.2 × 10^−7^ compared with OR = 0.58, 95% CI = 0.51–0.67, *P* = 1.4 × 10^−15^ for the minor allele G for the unconditional analysis (Figure 2)). Only HLA-B*15:01 showed statistically significant association after further conditioning on rs115625939 (OR = 0.76, 95%CI = 0.65-0.89, *P* = 5.1 × 10^−4^) (Table 2). The LD between rs115625939 and the other three SNPs is weak (*r*^2^ = 0.16 with rs9271898; *r*^2^ = 0 with rs2516448 and *r*^2^ = 0.01 with rs3130196, respectively) (Supplementary Table S1). The LD between the significant SNPs and classical HLA alleles is shown in Supplementary Table S1. Statistially significant associations were observed for rs9271898 (OR = 0.67, 95%CI = 0.61–0.74, *P* = 1.0 × 10^−15^), rs2516448 (OR = 1.25, 95% CI = 1.14–1.37, *P* = 6.1 × 10^−6^), rs3130196 (OR = 1.42, 95% CI = 1.27-1.59, *P* = 1.0 × 10^−9^) and rs115625939 (OR = 0.71, 95% CI = 0.60 – 0.85, *P* = 1.9 × 10^−4^) upon conditioning on significant HLA alleles/haplotypes, respectively (Supplementary Table S2). Collectively, these data provide evidence for a novel disease locus annotated by rs115625939 at 6p21.3, in addition to the three previously reported susceptibility loci in this region. The genetic variants that were highly correlated with each of the four independent loci (*r*^2^ ≥ 0.8) were grouped in bins and are shown in Supplementary Table S3. Similar associations were observed for the top signals when analyzing the CIN3 as a distinct group (Supplementary Table S4).

### Replication and combined results

A genotyping assay for replication of rs115625939 at 6p21.3 was not possible to design. Instead, a strong proxy of rs115625939, rs73730372 (*D’* = 1, *r*^2^ = 1), was successfully genotyped in the replication study, including an independent set of 956 cases (827 with CIN3 and 123 with cervical cancer) and 1,715 control subjects. The association between rs73730372 and CIN3 risk was independently replicated in the replication study (OR = 0.64, 95% CI = 0.54-0.77, *P* = 9.8 × 10^−7^ for the minor allele A) with no evidence of heterogeneity between two study phases (*P*-heterogeneity = 0.39). In contrast, no association was observed between rs73000875 at 18q12.3 (OR = 0.88, 95% CI = 0.69–1.11, *P* = 0.26 for the minor allele G) and risk of CIN3 in the replication series.

**Figure 3:**
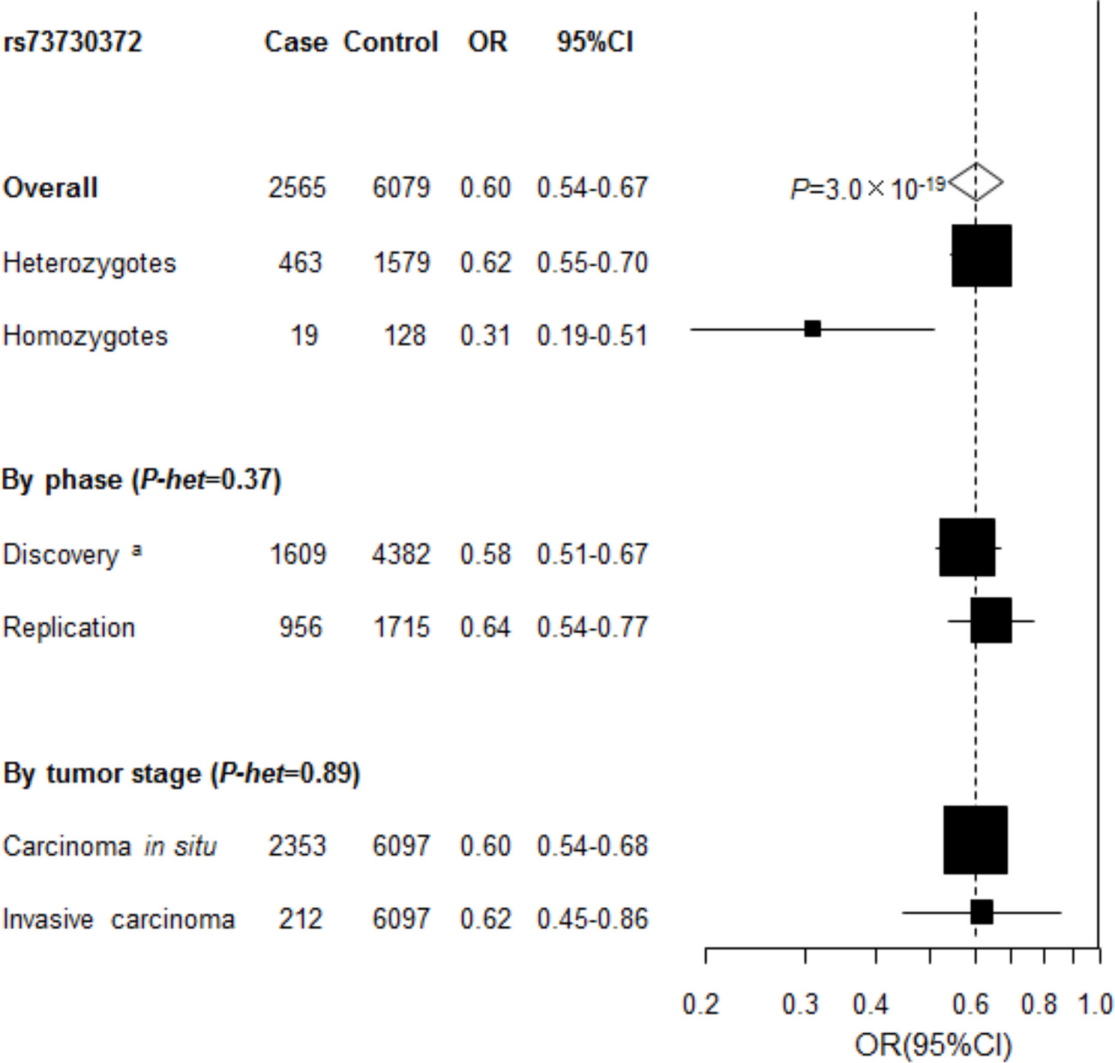
Association between rs73730372 and cervical disease in the combined series Unless specified, association between rs73730372, a proxy of rs115625939 (*D’*=1, *r*^2^ = 1) and cervical cancer in the combined data was estimated by logistic regression analysis for the minor allele in log-additive model with adjustment for study after pooling individual level data from the discovery and replication phase. P-het, P for heterogeneity, was derived from Cochran's *Q* test. a Derived from logistic regression analysis for the minor allele in log-additive model with adjustment for study and the one significant principal component generated by principal components analysis.

After pooling the discovery and replication data, the ORs (95% CI) were 0.62 (0.55–0.70) and 0.31(0.19 – 0.51) for heterozygous (genotype CT) and homozygous carriers (genotype TT) of rs73730372, compared to CC, respectively, yielding *P* = 3.0 **×** 10^−19^ for trend in the combined analysis (Figure 3). No heterogeneity for the association of rs73730372 was noted by tumor grade (CIN3 vs cervical cancer).

## DISCUSSION

We performed a pooled analysis of data from two GWASs by imputing over 10 million genetic variants and 424 classic HLA alleles. The results provided increased support for multiple independent susceptibility loci and classic HLA alleles within the MHC region, previously identified to be associated with cervical cancer. We have also provided support for the existence of a novel, independent, disease locus, as indicated by the SNP rs73730372 at 6p21.3. These findings suggest that immunological factors play an important role in the pathogenesis of CIN3. The risk or protective alleles may affect the binding affinity to high-risk HPVs or expression level of HLA molecules, hence influence susceptibility to infection and/or persistence of high-risk HPVs.

The minor allele of rs73730372 was associated with a 40% reduced risk of CIN3 with the homozygous genotype conferring a 69% reduced risk (Figure 2). This is one of the strongest common genetic protective variants identified CIN3 SNP rs73730372 correlates (*r*^2^ ≥ 0.9) with 16 SNPs. It is located 11 kb upstream of *HLA-DQA1* and 45 kb downstream of *HLA-DQB1*. rs73730372 has recently been identified as expression quantitative trait locus (eQTL) that influence the expression of both *HLA-DQA1* and *HLA-DQB1* in blood [4]. *HLA-DQA1* and *HLA-DQB1* belong to the HLA class II alpha and beta chain paralogs, respectively. Together, their gene products form a protein complex called an antigen-binding DQαβ heterodimer. This complex presents foreign peptides to the immune system to trigger the body's immune response, and therefore play a key role in the immune recognition process and subsequent clearance of virally-infected cells. Class II molecules are expressed in antigen presenting cells (APC: B Lymphocytes, dendritic cells, macrophages) and are known to be important in the regulation of the immune response to viral and other infections. Class II molecules present antigenic peptides to CD4^+^ T helper cells to initiate a cell-mediated immune response [5]. Our study points to the importance of expression level of *HLA-DQA1* and *HLA-DQB1* for the susceptibility to develop cervical carcinoma. Impaired class II gene expression has been reported in genital HPV infections and in lesions caused by HPV [6–8]. The increased incidence and progression of HPV infections in immunosuppressed individuals illustrates the critical importance of the CD4^+^ T-cell-regulated cell-mediated immune response in the resolution of HPV infection [5, 9]. Failure to develop an effective cell-mediated immune response to clear infection results in a persistent infection and, in the case of the oncogenic HPVs, an increased probability of progression to CIN3 and cervical cancer [9]. The minor allele G also shows lower affinity of binding forkhead box L1 (Foxl1) and homeobox D10 (Hoxd10) [10] proteins. Foxl1 encodes a member of the forkhead/winged helix-box (FOX) family of transcription factors, which play a critical role in the regulation of multiple processes including metabolism, cell proliferation and gene expression during ontogenesis. Hoxd10 is a member of the Abd-B homeobox family and encodes a protein with a homeobox DNA-binding domain. Recent studies suggest that HoxD10 functions as a candidate tumor suppressor [11, 12].

A GWAS of cervical cancer (CIN2, CIN3 and cervical cancer) based on patients of mixed European descent identified associations with both *HLA*-B*07 and HLA-B*15:01 and squamous cervical cancer (personal communication). Consistent with this, we found associations of CIN3 (all squmous cell carcinoma) with both *HLA*-B*07:02 and HLA-B*15:01 in our study. In addition, we confirmed associations with *HLA*-DRB1*15:01 and HLA-DQB1*06:02 as well as HLA-DRB1*13:01, HLA-DQA1*01:03 and HLA-DQB1*06:03, which are usually reported to be associated with risk of cervical cancer [13–15]. We also identified an association of CIN3 with HLA-C*07:02.

Another GWAS performed in the Chinese population reported invasive cervical carcinoma to be associated with variants in the HLA-DP region (rs4282438 and rs9277952), and with two non-MHC loci: Exocyst complex component 1 (*EXOC1*) at 4q12 (rs13117307) and (Gasdermin B (*GSDMB*) at 17q12 (rs8067378) [16]. As shown in Supplementary Table S5, none of the loci reported in this GWAS showed evidence of association in our study. The lack of success in replicating the effect of these variants could be attributed to a number of factors An important confounder is probably the different ethnic backgrounds between the Asian (Chinese) and the Caucasian (Swedish) population. The clinical endpoint also differed between these two studies, from CIN3 to cervical cancer. The differences in association seen between our study and that of the Chinese population could also be due to variability in the frequency of HPV types, intratype variation, or host-virus interactions. Since we lack information on the infecting HPV for individual cases in our study, we are unable to take this into consideration in the analyses.

Several limitations should be noted in the present study. First, our results apply to CIN3, which represents the vast majority of cases. We therefore have higher power to identify variants associated with CIN3 and a limited power to detect association with cervical cancer. Additional risk factors may be involved in progression from CIN3 to cervical cancer. However, CIN3 and cervical cancer share the same main etiological factor, namely persistent infection with oncogenic HPVs [2], indicating that the genetic susceptibility loci identified for CIN3 may have a similar effect on the two cancer stages. Second, HPV status is not available in the present study, thus our findings could relate to some behavioral characteristics associated with HPV acquisition.

In summary, by pooling data from two GWASs and imputing over 10 million genetic variants, we have been able to comprehensively examine the association of CIN3 risk with common variants, rare variants and classical HLA alleles. This study strengthened the evidence for previously postulated susceptibility loci and provided support for a novel susceptibility locus for CIN3. The present study also provides an important proof-of-principle that the 1000 Genomes imputation can be used to detect novel, low frequency-large effect associations, thereby extending the utility of pre-existing GWAS data.

## MATERIALS AND METHODS

### Study population

The SNP data of the first GWAS was generated using the Illumina Omni Express BeadChip (Illumina, San Diego, CA) (731,422 SNPs) in the Swedish population. The details of population and QC have been described elsewhere [17]. Briefly, after stringent quality control, data from 1,034 cervical disease patients (971 with CIS and 63 with invasive carcinoma) and 3,948 control subjects were available for 632,668 SNPs, with an overall call rate of 99.92%. The second GWAS data set, also from the Swedish population, was generated using the Affymetrix Genome-wide Human SNP array 5.0 with 440,794 SNP markers. After systematic QC steps, data from 616 case subjects (597 with CIN3 and 19 with cervical cancer) and 506 control subjects were available for 341,358 SNPs with an overall call rate of 99.79% [17].

Details on study design and subjects recruitment of the replication study have been described previously [18]. Briefly, the replication study consisted of 961 patients (827 with CIN3 and 123 cervical cancer) and 1,725 matched healthy controls from the Swedish population. Informed consent was obtained from all subjects, and each study was approved by the regional ethical review board. All the cases in the discovery and replication studies are squamous cell carcinoma.

### SNP imputation, two GWASs merging and quality control

The details of imputation have been described previously [17]. In brief, the second GWAS data set was changed to hg19 from hg18 using the USCS LiftOver tool (http://genome.sph.umich.edu/wiki/LiftOver). Both GWAS data sets were separately phased using the shapeit-tool (https://mathgen.stats.ox.ac.uk/genetics_software/shapeit/shapeit.html) (19). They were then separately split into chunks of approximate 5Mb containing at least 200 genotyped SNPs. No chunks spanned the centromeres. Genetic variants across each chunk not genotyped on the Ilumina HumanOmniExpress BeadChip or Affymetrix Genome-wide Human SNP arrays 5.0 were imputed, respectively, using the program IMPUTE2 [20] with phased haplotypes in subjects from the December 2013 release of the 1000 Genomes Project data (https://mathgen.stats.ox.ac.uk/impute/impute_v2.html#reference) [21] as a scaffold. Imputed variants with info-score < 0.3 in either data set were removed. The two data sets were then merged and markers with less than 0.9 in posterior genotypic probability in more than 5% of the samples or monomorphic were removed. Finally, genotypes were called from posterior probabilities using 0.9 as threshold using gtool (http://www.well.ox.ac.uk/~cfreeman/software/gwas/gtool.html). Further QC was applied to the merged data set. The exclusion criteria for genetic variants were: (1) genotype frequency that deviated from Hardy-Weinberg Equilibrium (HWE) among control subjects (*P* < 1 × 10^−7^), and (2) a statistically significant difference in minor allele frequency (MAF) between the two GWASs (*P* < 1 × 10^−7^). As shown in Supplementary Table S6, unexpected duplicates (11 subjects) and first-degree and second-degree relatives (9 subjects) were removed based on identity-by-state (IBS) estimates calculated in PLINK [22]. Utilizing a set of 15,872 SNPs genotyped in both GWASs and evenly distributed across the genome in low linkage disequilibrium (LD) (pair-wise *r*^2^ < 0.02), a principal components analysis (PCA) using the EIGENSTRAT software [23] excluded 8 additional samples detected as outliers.

### Genotyping and quality control

A genotyping assay for rs115625939 was not possible to design. To validate the findings from the whole-genome scan, SNP rs73730372 was genotyped for replication as a proxy for rs115625939 (*D’* = 1, *r*^2^ = 1.00) with template-directed dye-terminator incorporation with fluorescence-polarization detection (FP-TDI) (Tecan, Männedorf, Switzerland). SNP rs73000875 was also genotyped using the same assay. Eight percent of the samples were selected for repeat genotyping as duplicates, yielding a reproducibility of 100%. Genotype success rate was greater than 98.7% and genotype distributions were consistent with that expected by HWE for each SNP.

### Statistical analysis

In the discovery phase, the potential for population stratification was investigated by PCA undertaken with the EIGENSTRAT package [23]. One significant PC derived from PCA based on the Tracy-Widom statistic (*P* < 0.05) was statistically significantly associated with case-control status (*P* < 0.05). Adjustment for population stratification was performed by including significant PC as covariate in the logistic regression. The association between each genetic variant and risk of cervical disease was estimated by the OR per minor allele and 95% CI using multivariate unconditional logistic regression, assuming a log-additive model with adjustment for study and the significant PC with PLINK. Conditioning analysis on significant HLA alleles/haplotypes was performed in PLINK using logistic regression in log-additive model conditioning on B*07:02-C*07:02, B*15:01, DRB1*13:01-DQA1*01:03-DQB1*06:03 and DRB1*15:01-DQB1*06:02 with adjustment for study and one significant principal component generated by principal components analysis. The conditioning HLA alleles/haplotypes were entered into the model simply as covariates.

In the replication study, the association between each genetic variant and risk of cervical disease was estimated by the OR per minor allele and 95% CI using unconditional logistic regression assuming a log-additive model. Individual level data from the discovery and replication phase were pooled, making for a total of 2,565 cervical cases and 6,079 controls. In the combined analysis, association between each genetic variant and risk of cervical disease was estimated by the OR per minor allele and 95% CI using unconditional logistic regression assuming a log-additive model with adjustment for study. We then conducted stratified analysis by study phase and tumor grade. Heterogeneity of ORs was assessed using the Cochran's Q test. Results that obtained a level of significance of a *P* < 5.0 × 10^−8^ were considered statistically significant at the genome-wide level. All replication and combined analyses were conducted using SAS 9.3 software. All statistical tests were two-sided.

### Imputation at classical HLA alleles and analysis

The T1DGC panel contains 5,868 SNPs (genotyped with Illumina Immunochip) and 4-digit classical HLA types for *HLA-A*, -*B*, -*C*, -*DPB1*, -*DQA1*, -*DQB1* and -*DRB1* for 5,225 unrelated individuals (10,450 haplotypes) [24]. With the T1DGC reference panel, we imputed 126 classical 2-digit alleles and 298 classical 4-digit alleles at *HLA-A*, *-B*, *-C*, -*DPB1, -DQA1*, -*DQB1* and -*DRB1* using SNP2HLA [24]. Unconditional logistic regression using the imputed genotype for each classical HLA allele was carried out using PLINK with adjustment for study and the significant PC identified from PCA.

### Genotyping of classical HLA loci

The typing of *HLA-A, -B*, *-DRB1, -DQB1* and *-DPB1* in 254 cervical cancer patients included in the present study was previously performed [25] by PCR amplification of groups of alleles using biotinylated PCR primers, followed by hybridization to immobilized sequence-specific oligonucleotide probes in a linear-array format. Genotypes were determined using a computer algorithm on the basis of the pattern of sequence-specific oligonucleotide-probe hybridization.

## SUPPLEMENTARY FIGURES AND TABLES


