# Supplementary material for: Pooled analysis of genome-wide association studies of cervical intraepithelial neoplasia 3 (CIN3) identifies a new susceptibility locus
Source: Oncotarget. 2016 Jun 7;7(27):42216–24. doi: 10.18632/oncotarget.9916 (PMC5173129; doi:10.18632/oncotarget.9916)
Supplement: Supplementary file 1 [file oncotarget-07-42216-s001.pdf]

Pooled analysis of genome-wide association studies of cervical intraepithelial neoplasia 3 (CIN3) identifies a new susceptibility locus

Supplementary Materials

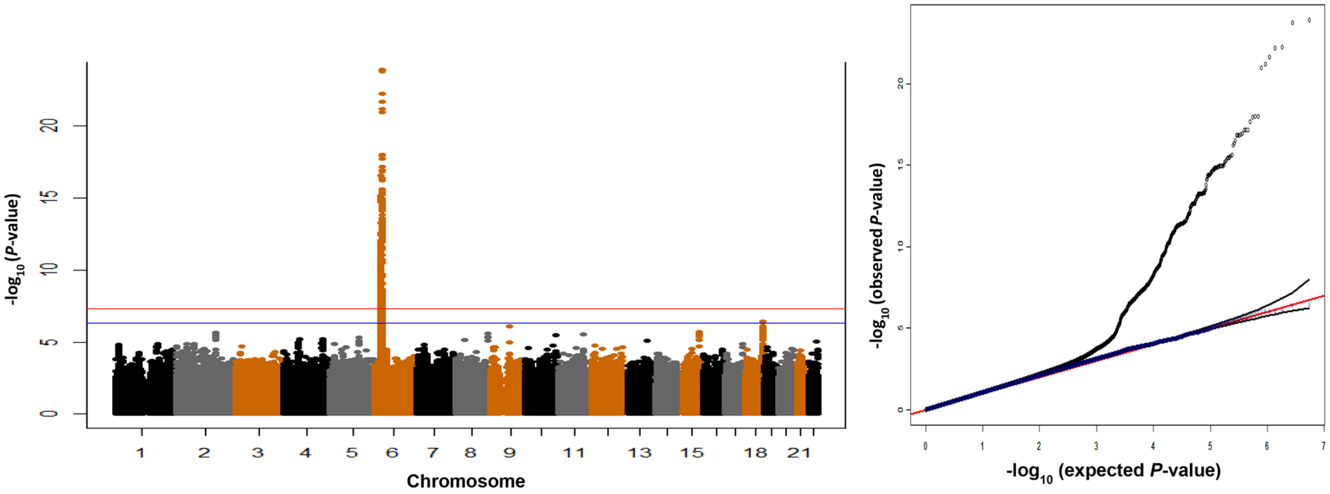

Supplementary Figure S1: Genome-wide association results for cervical precancer. (**A**) Manhattan plot of *P* values. The horizontal red line and blue line represent $P = 5 \times 10^{-8}$ and $P = 5 \times 10^{-7}$, respectively. (**B**) Quantile-quantile plot of the expected versus observed -log10 (P value) distribution in the genome-wide association analysis. A quantile-quantile plot of the log-additive model is shown in black ($\lambda = 1.08$) for all the SNPs that pass quality control. Additional quantile-quantile plot, which excludes all SNPs in the extended MHC region of association, is superimposed in blue ($\lambda = 1.02$). The line of equality is indicated in red. The black line surrounding it corresponds to 95% confidence interval which is formed by calculating, for each statistic, the 2.5th and 97.5th centiles of the distribution of the order statistic under random sampling and the null hypothesis. In general, the plotted values fall into the 95% confidence interval, suggesting that there is no significant inflation of the test statistic for the SNPs.

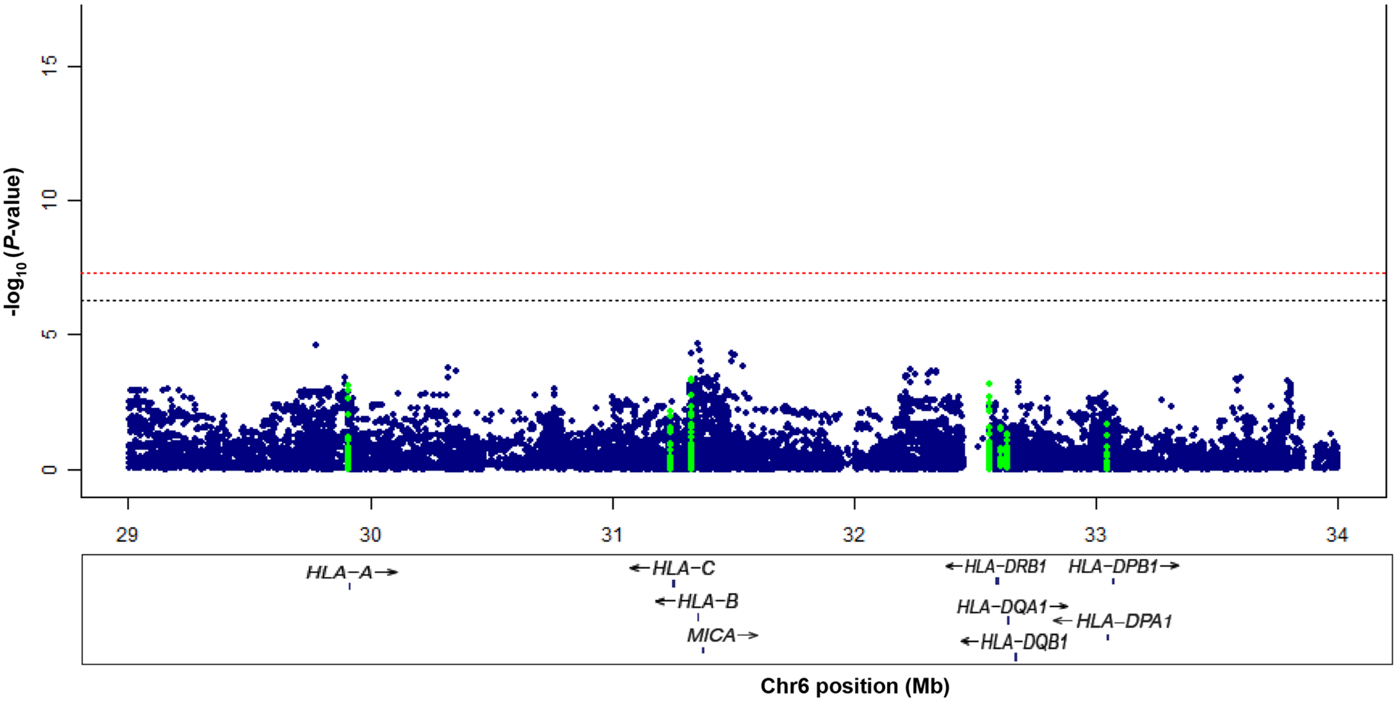

Supplementary Figure S2: Association results upon conditioning on rs9271898, rs2516448, rs3130196 and rs73730372 at 6p21.3. The horizontal red line and blue line represent $P = 5 \times 10^{-8}$ and $P = 5 \times 10^{-7}$, respectively.

Supplementary Table S1: Linkage disequilibrium (*D'* and r^2) between the top signals

D' / r^2	rs9271898	rs2516448	rs3130196	rs115625939	*B*07:02*	*B*15:01*	*C*07:02*	*DRB1*15:01*	*DRB1*13:01*	*DQA1*01:03*	*DQB1*06:02*	*DQB1*06:03*
rs9271898	—	0.020	0.050	0.940	0.483	0.329	0.481	1.0	0.982	0.937	0.985	0.903
rs2516448	0	—	0.076	0.010	0.988	0.987	0.855	0.462	0.293	0.302	0.496	0.296
rs3130196	0	0.001	—	0.120	0.002	0.143	0.012	0.236	0.005	0.012	0.235	0.001
rs115625939	0.16	0	0.010	—	0.411	0.052	0.475	1.0	0.989	0.962	0.941	0.941
*B*07:02*	0.039	0.172	0	0.005	—	1.0	0.959	0.497	0.310	0.320	0.492	0.291
*B*15:01*	0.013	0.126	0	0.002	0.023	—	1.0	0.503	0.140	0.130	0.556	0.142
*C*07:02*	0.040	0.136	0	0.007	0.872	0.024	—	0.466	0.334	0.344	0.479	0.317
*DRB1*15:01*	0.173	0.039	0.001	0.032	0.236	0.006	0.215	—	—	1.0	0.988	0.534
*DRB1*13:01*	0.090	0.007	0	0.494	0.001	0.013	0.002	—	—	0.992	1.0	0.988
*DQA1*01:03*	0.085	0.008	0	0.480	0.002	0.012	0.002	0.016	0.958	—	1.0	0.963
*DQB1*06:02*	0.163	0.044	0.001	0.027	0.239	0.007	0.220	0.947	0.015	0.016	—	—
*DQB1*06:03*	0.080	0.008	0	0.467	0.001	0.014	0.002	0.005	0.933	0.910	—	—

D' values are given above the diagonal; r^2 values are given below the diagonal.

Supplementary Table S2: Association results of 4 SNPs at 6p21.3 upon conditioning on significant HLA alleles/haplotypes

		Single SNP[a]		Conditioning on significant HLA alleles/haplotypes[b]	
**Loci**	**SNP**	**OR (95%CI)**	***P***	**OR (95%CI)**	***P***
1	rs9271898	0.64 (0.59–0.70)	1.2×10^{-24}	0.67 (0.61–0.74)	1.0×10^{-15}
2	rs2516448	1.39 (1.28–1.52)	1.1×10^{-15}	1.25 (1.14–1.37)	6.1×10^{-6}
3	rs3130196	1.40 (1.26–1.57)	2.3×10^{-9}	1.42 (1.27–1.59)	1.0×10^{-9}
4	rs115625939	0.58 (0.51–0.67)	1.4×10^{-15}	0.71 (0.60–0.85)	1.9×10^{-4}

MAF, minor allele frequency; OR, odds ratio; CI, confidence interval; *P*, two-sided *P* value corresponding to the OR.
[a]ORs and 95%CIs for minor allele in log-additive model were derived from logistic regression with adjustment for study and one significant principal component generated by principal components analysis.
[b]Derived from logistic regression in log-additive model conditioning on *B*07:02-C*07:02, B*15:01, DRB1*13:01-DQA1*01:03-DQB1*06:03* and *DRB1*15:01-DQB1*06:02* with adjustment for study and one significant principal component generated by principal components analysis.

Supplementary Table S3: Association results of variants in each bin with risk of cervical precancer

				MAF		Association[b]	
**Variants**	**Position[a]**	**r^2**	**Alleles**	**Case**	**Control**	**OR (95%CI)**	***P***
bin1							
rs9271898	32595972	1.00	G/A	0.38	0.49	0.64 (0.59−0.70)	1.20×10^{-24}
rs9271897	32595954	1.00	G/A	0.38	0.49	0.64 (0.59−0.70)	1.65×10^{-24}
rs9272143	32600803	0.99	T/C	0.39	0.49	0.66 (0.61−0.72)	4.2×10^{-22}
rs9272044	32598971	0.99	C/A	0.39	0.48	0.66 (0.61−0.72)	6.13×10^{-22}
rs9272067	32599401	0.98	C/A	0.39	0.48	0.66 (0.61−0.72)	1.04×10^{-21}
rs116603449	32595194	0.98	C/T	0.39	0.49	0.66 (0.61−0.72)	5.54×10^{-23}
rs9272120	32600501	0.98	A/G	0.39	0.49	0.66 (0.61−0.72)	6.04×10^{-23}
rs9272130	32600638	0.97	A/G	0.39	0.49	0.66 (0.61−0.72)	2.12×10^{-22}
rs9272132	32600677	0.84	A/G	0.55	0.47	1.39 (1.28−1.51)	2.25×10^{-15}

rs9272114	32600317	0.83	T/C	0.55	0.46	1.42 (1.31−1.54)	5.85×10^{-17}
rs9272043	32598946	0.82	T/G	0.55	0.47	1.39 (1.28−1.51)	2.37×10^{-15}
rs9272105	32599999	0.81	G/A	0.54	0.46	1.40 (1.29−1.52)	5.88×10^{-16}
rs9272254	32603098	0.81	C/T	0.54	0.46	1.40 (1.29−1.52)	4.69×10^{-16}
rs9272070	32599485	0.81	T/G	0.54	0.46	1.39 (1.28−1.50)	4.27×10^{-15}
rs9272229	32602568	0.81	C/T	0.54	0.46	1.41 (1.30−1.53)	2.48×10^{-16}
bin2							
rs2516448	31390410		A/G	0.42	0.50	0.72 (0.66−0.78)	1.14×10^{-15}
rs36002510	31388175	1.00	-/T	0.42	0.50	0.72 (0.66−0.78)	1.23×10^{-15}
rs2596528	31385485	1.00	C/T	0.42	0.50	0.72 (0.66−0.78)	1.31×10^{-15}
rs2596530	31387373	1.00	G/A	0.42	0.50	0.72 (0.66−0.78)	1.20×10^{-15}
rs2596531	31387557	1.00	C/T	0.42	0.50	0.72 (0.66−0.78)	1.20×10^{-15}
rs2853981	31378768	1.00	T/C	0.42	0.50	0.72 (0.66−0.78)	1.83×10^{-15}
rs2853977	31379304	1.00	T/A	0.42	0.50	0.72 (0.66−0.78)	1.82×10^{-15}
rs67841474	31380160	1.00	-/G	0.42	0.50	0.72 (0.66−0.78)	1.99×10^{-15}
rs2256174	31380422	1.00	A/G	0.42	0.50	0.72 (0.66−0.78)	1.40×10^{-15}
rs2256183	31380529	1.00	A/G	0.42	0.50	0.72 (0.66−0.78)	1.40×10^{-15}
rs2844511	31389784	1.00	T/C	0.42	0.50	0.72 (0.66−0.78)	1.34×10^{-15}
rs1882	31382911	0.90	A/G	0.40	0.47	0.73 (0.67−0.79)	4.99×10^{-14}
bin3							
rs3130196	33063219	1.00	T/C	0.17	0.13	1.40 (1.26−1.57)	2.29×10^{-9}
rs3117209	33066262	1.00	C/T	0.17	0.13	1.40 (1.26−1.57)	2.84×10^{-9}
rs3130214	33074712	1.00	G/C	0.17	0.13	1.40 (1.26−1.57)	2.85×10^{-9}
rs3130217	33078020	1.00	C/T	0.17	0.13	1.39 (1.24−1.56)	6.83×10^{-9}
rs3130218	33078163	1.00	G/A	0.17	0.13	1.39 (1.25−1.56)	6.10×10^{-9}
rs3117030	33089316	0.94	A/G	0.16	0.12	1.38 (1.24−1.55)	1.49×10^{-9}
rs3139278	33092412	0.94	C/T	0.16	0.12	1.38 (1.24−1.55)	1.60×10^{-8}
rs146072057	33092862	0.94	-/A	0.16	0.12	1.38 (1.24−1.55)	1.56×10^{-8}
rs3129376	33093309	0.94	C/T	0.16	0.12	1.38 (1.24−1.55)	1.60×10^{-8}
rs9282434	33094058	0.94	-/T	0.16	0.12	1.38 (1.23−1.54)	2.07×10^{-8}
rs3117018	33094720	0.93	T/C	0.16	0.12	1.38 (1.23−1.54)	1.91×10^{-8}
r3117015	33095555	0.93	A/T	0.16	0.12	1.38 (1.24−1.55)	1.61×10^{-8}
rs9277764	33095762	0.93	A/G	0.16	0.12	1.38 (1.24−1.55)	1.61×10^{-8}
rs3129271	33096714	0.93	G/C	0.16	0.12	1.38 (1.24−1.55)	1.61×10^{-8}
rs3117003	33096777	0.93	C/T	0.16	0.12	1.38 (1.24−1.55)	1.61×10^{-8}
rs3116993	33098828	0.93	C/T	0.16	0.12	1.38 (1.23−1.54)	2.12×10^{-8}
rs3129270	33097423	0.93	G/A	0.16	0.12	1.39 (1.24,1.55)	1.29×10^{-8}
rs3116992	33099129	0.93	C/T	0.16	0.12	1.38 (1.23−1.54)	2.08×10^{-8}
rs3130239	33101269	0.92	G/A	0.16	0.12	1.39 (1.24−1.55)	1.23×10^{-8}
rs3129262	33102004	0.92	C/T	0.16	0.12	1.39 (1.24−1.55)	1.23×10^{-8}
rs3116976	33111953	0.92	C/T	0.16	0.12	1.39 (1.24−1.55)	1.35×10^{-8}
rs3129225	33113055	0.92	C/G	0.16	0.12	1.39 (1.24−1.55)	1.35×10^{-8}
rs3116974	33114333	0.92	T/C	0.16	0.12	1.39 (1.24−1.55)	1.35×10^{-8}
rs3116965	33120677	0.92	A/G	0.16	0.12	1.39 (1.24−1.55)	1.39×10^{-8}
rs3116961	33124031	0.92	G/A	0.16	0.12	1.38 (1.24−1.55)	1.49×10^{-8}
rs3130169	33048102	0.92	G/A	0.16	0.12	1.39 (1.24−1.56)	1.43×10^{-8}

rs3128959	33048380	0.92	G/A	0.16	0.12	1.40 (1.25−1.57)	1.23×10^{-8}
rs3097670	33046752	0.92	G/C	0.16	0.12	1.39 (1.24−1.56)	1.46×10^{-8}
rs3129203	33127757	0.91	G/A	0.16	0.13	1.37 (1.23−1.54)	2.70×10^{-8}
rs3116989	33099538	0.87	A/C	0.17	0.13	1.33 (1.19−1.49)	3.63×10^{-7}
rs55954100	33099998	0.86	-/A	0.17	0.13	1.33 (1.20−1.49)	2.92×10^{-7}
rs2092305	33100189	0.86	T/C	0.17	0.13	1.33 (1.20−1.49)	2.92×10^{-7}
rs3130161	33125858	0.86	A/C	0.17	0.13	1.33 (1.19−1.49)	3.47×10^{-7}
rs3130159	33124693	0.86	G/C	0.17	0.13	1.33 (1.19−1.48)	4.61×10^{-7}
rs2744505	33153122	0.80	G/C	0.18	0.14	1.37 (1.23−1.53)	7.33×10^{-9}
rs71709900	33076147	0.80	-/TCC	0.19	0.15	1.34 (1.21−1.49)	3.61×10^{-8}
bin4							
rs115625939	32583584	1.00	A/G	0.09	0.15	0.58 (0.51−0.66)	1.39×10^{-15}
rs73730372	32584581	1.00	C/T	0.09	0.15	0.58 (0.51−0.67)	3.05×10^{-15}
rs73730377	32584880	1.00	A/G	0.09	0.15	0.59 (0.51−0.67)	3.72×10^{-15}
rs508318	32584894	1.00	C/A	0.09	0.15	0.59 (0.51−0.67)	3.72×10^{-15}
rs113016235	32584977	1.00	T/G	0.09	0.15	0.59 (0.51−0.67)	3.72×10^{-15}
rs113243185	32585071	1.00	G/C	0.09	0.15	0.59 (0.52−0.67)	6.93×10^{-15}
rs113705304	32590453	0.96	C/G	0.09	0.14	0.58 (0.51−0.67)	3.96×10^{-15}
rs34831921	32590768	0.96	G/T	0.09	0.14	0.58 (0.51−0.67)	3.96×10^{-15}
rs34376179	32591238	0.96	T/C	0.09	0.14	0.57 (0.50−0.66)	1.10×10^{-15}
rs34967069	32591248	0.96	G/A	0.09	0.14	0.57 (0.50−0.66)	1.10×10^{-15}
rs73728618	32591567	0.96	T/A	0.09	0.14	0.57 (0.50−0.66)	1.10×10^{-15}
rs35001273	32591664	0.96	C/T	0.09	0.14	0.57 (0.50−0.66)	1.10×10^{-15}
rs35867827	32592068	0.93	A/G	0.08	0.14	0.58 (0.50−0.67)	1.56×10^{-14}
rs35224566	32592313	0.93	A/G	0.08	0.14	0.57 (0.50−0.66)	7.58×10^{-15}
rs34518860	32594103	0.96	G/A	0.09	0.14	0.57 (0.50−0.66)	2.95×10^{-15}
rs116518618	32594998	0.96	C/T	0.09	0.14	0.57 (0.50−0.66)	1.07×10^{-15}
rs28584179	32626119	0.96	C/T	0.08	0.14	0.57 (0.49−0.65)	1.77×10^{-15}

MAF, minor allele frequency; OR, odds ratio; CI, confidence interval; *P*, two-sided *P* value corresponding to the OR.
[a]GRCh37/hg19 Assembly.
[b]ORs and 95%CIs for minor allele in log-additive model were derived from logistic regression with adjustment for study and one significant principal component generated by principal components analysis.

Supplementary Table S4: Comparison of association results of top signals between CIN3+cervical cancer and CIN3

	CIN3+cervical cancer		CIN3		
	**OR (95%)[a]**	***P*[a]**	**OR (95%)[a]**	***P*[a]**	**P_{-het}[b]**
**SNP**					
rs9271898	0.64 (0.59−0.70)	1.2×10^{-24}	0.65 (0.59−0.70)	1.3×10^{-23}	0.80
rs2516448	1.39 (1.28−1.52)	1.1×10^{-15}	1.39 (1.28−1.52)	1.2×10^{-14}	1.0
rs3130196	1.40 (1.26−1.57)	2.3×10^{-9}	1.43 (1.28−1.60)	4.4×10^{-10}	0.79
rs115625939	0.58 (0.51−0.67)	1.4×10^{-15}	0.59 (0.51−0.67)	1.7×10^{-14}	0.86

**HLA-B**					
*HLA_B*0702*	1.41 (1.27−1.57)	1.4×10^{-10}	1.39 (1.25−1.55)	1.0×10^{-9}	0.85
*HLA_B*1501*	0.67 (0.58−0.77)	2.6×10^{-8}	0.67 (0.59−0.78)	9.9×10^{-8}	1.0
**HLA-C**					
*HLA_C*0702*	1.37 (1.24−1.52)	2.6×10^{-9}	1.36 (1.22−1.51)	1.7×10^{-8}	0.92
**HLA-DRB1**					
*HLA_DRB1*1301*	0.49 (0.40−0.59)	1.8×10^{-13}	0.49 (0.40−0.59)	2.9×10^{-13}	1.0
*HLA_DRB*1501*	1.36 (1.22−1.51)	9.7×10^{-9}	1.34 (1.21−1.49)	6.5×10^{-8}	0.85
**HLA-DQA1**					
*HLA_DQA1*0103*	0.49 (0.40−0.59)	5.6×10^{-14}	0.48 (0.40−0.58)	9.8×10^{-14}	0.88
**HLA-DQB1**					
*HLA_DQB1*0603*	0.54 (0.45−0.64)	1.5×10^{-11}	0.54 (0.45−0.65)	2.9×10^{-11}	1.0
*HLA_DQB1*0602*	1.32 (1.19−1.47)	2.5×10^{-7}	1.30 (1.17−1.45)	1.7×10^{-6}	0.84

OR, odds ratio; CI, confidence interval; *P*, two-sided *P* value corresponding to the OR; P_{-het}, *P* for heterogeneity.

[a]ORs and 95%CIs for minor allele in log-additive model were derived from logistic regression with adjustment for study and one significant principal component generated by principal components analysis.

[b] *P* for heterogeneity was evaluated using the Cochran Q test statistic.

Supplementary Table S5: Association of the top hits identified by Chinese GWAS of cervical cancer in the present data

CHR	SNP	Position[a]	Nearby gene	OR(95%CI)[b]	*P*
6	rs9277952	33204274	*HLA-DP*	0.97 (0.83−1.13)	0.69
6	rs4282438	33072172	*HLA-DP*	1.10 (0.88−1.36)	0.41
4	rs13117307	56751740	*EXOC1*	1.08 (0.98−1.19)	0.12
17	rs8067378	38051348	*GSDMB*	0.91 (0.83−0.99)	0.03

OR, odds ratio; CI, confidence interval; *P*, two-sided *P* value corresponding to the OR.

[a]GRCh37/hg19 Assembly.

[b]ORs and 95%CIs for minor allele in log-additive model were derived from logistic regression with adjustment for study and one significant principal component generated by principal components analysis.

Supplementary Table S6: Exclusion criteria of subjects for GWAS

Sample	Cases	Controls	Total
**All**	**1,650**	**4,454**	**6,104**
**Exclusion criteria for subjects:**			
Unexpected duplicates	11	0	11
Unexpected 1st and 2nd degree relatives	4	5	9
Population outliers	1	7	8
**Included in analysis**	**1,634**	**4,442**	**6,076**
